# Lung Infection Segmentation for COVID-19 Pneumonia Based on a Cascade Convolutional Network from CT Images

**DOI:** 10.1155/2021/5544742

**Published:** 2021-04-15

**Authors:** Ramin Ranjbarzadeh, Saeid Jafarzadeh Ghoushchi, Malika Bendechache, Amir Amirabadi, Mohd Nizam Ab Rahman, Soroush Baseri Saadi, Amirhossein Aghamohammadi, Mersedeh Kooshki Forooshani

**Affiliations:** ^1^Department of Telecommunications Engineering, Faculty of Engineering, University of Guilan, Rasht, Iran; ^2^Faculty of Industrial Engineering, Urmia University of Technology, Urmia, Iran; ^3^School of Computing, Faculty of Engineering and Computing, Dublin City University, Ireland; ^4^Department of Electrical Engineering, Islamic Azad University, South Tehran Branch, Tehran, Iran; ^5^Department of Mechanical and Manufacturing Engineering, Faculty of Engineering and Built Environment, Universiti Kebangsaan Malaysia, 43600 Bangi Selangor, Malaysia; ^6^Institute of Visual Informatics, Universiti Kebangsaan Malaysia, Bangi, Selangor, Malaysia; ^7^Department of Electronics and Telecommunications, Polytechnic University, Turin, Italy

## Abstract

The COVID-19 pandemic is a global, national, and local public health concern which has caused a significant outbreak in all countries and regions for both males and females around the world. Automated detection of lung infections and their boundaries from medical images offers a great potential to augment the patient treatment healthcare strategies for tackling COVID-19 and its impacts. Detecting this disease from lung CT scan images is perhaps one of the fastest ways to diagnose patients. However, finding the presence of infected tissues and segment them from CT slices faces numerous challenges, including similar adjacent tissues, vague boundary, and erratic infections. To eliminate these obstacles, we propose a two-route convolutional neural network (CNN) by extracting global and local features for detecting and classifying COVID-19 infection from CT images. Each pixel from the image is classified into the normal and infected tissues. For improving the classification accuracy, we used two different strategies including fuzzy *c*-means clustering and local directional pattern (LDN) encoding methods to represent the input image differently. This allows us to find more complex pattern from the image. To overcome the overfitting problems due to small samples, an augmentation approach is utilized. The results demonstrated that the proposed framework achieved precision 96%, recall 97%, *F* score, average surface distance (ASD) of 2.8 ± 0.3 mm, and volume overlap error (VOE) of 5.6 ± 1.2%.

## 1. Introduction

Since December 2019, the world has been experiencing a new disease caused by SARS-CoV-2, which can cause asthma symptoms, acute respiratory malfunctioning, and even permanent changes to the biology of the lungs in patients regardless of their age limit. This disease was reported for the first time in Wuhan, Hubei province of China, and became a pandemic all over the world [1, 2]. The common symptoms of COVID-19 are shortness of breath, diarrhoea, coughing, sore throat, headaches, and fever. Vanishing of taste, nasal blockage, loss of smell, aches, and tiredness can also be observed in patients. The new infectious disease caused by the virus was named Coronavirus Disease 2019 (COVID-19) by the World Health Organization (WHO), and this coronavirus was named as SARS-CoV-2 by the International Committee on Taxonomy of Viruses (ICTV) [3, 4]. As there are only some definite vaccines available to prevent COVID-19, most of the unvaccinated people can be easily infected. One of the best ways to prevent the spread of virus infection in healthy persons is isolation and diagnosis of the infected person by any possible legal approach. One of the best methods is through the X-ray or CT images of patients' chest [5–7].

Inflammation growths in the lung can pose significant risks to human health. The increasing occurrence of infected people among the population demands more effective treatments along with a cost-efficient procedure that relies on its primary diagnosis. Providing prompt and precise recognition of the infected tissue plays a key role in effective patient treatment and survival [8, 9].

A CT scan or computed tomography scan as a routine tool and a high sensitivity for the diagnosis of COVID-19 are broadly employed in hospitals and can perform early screening for the defected tissue to recognize them precisely [10–12]. Doctors and specialists are increasingly employing such imaging modality to categorize local injuries and lesions [13]. Also, due to intensity similarity between lesions and normal tissues in CT images, the precise detection and segmentation of the infected area are certainly a cumbersome task, even for experienced radiologists or doctors [14, 15]. The flow of detection and feature extraction of texture information from the lung via manual observation is a time-consuming, tedious, and monotonous process. Computer-aided diagnostic (CAD) approaches are used for such tasks and are based on artificial intelligence and machine learning algorithms to recognize the border differences between two objects. These procedures are standardizable, reproducible, and can be useful in enhancing diagnostic accuracy in a very short time. These procedures act by helping doctors and experts to accomplish accurately sophisticated tasks, employing a combination of diversity classification approaches with a practical running time [3, 16].

Image segmentation is a complex and challenging area of the biomedical engineering task that is affected by numerous aspects, including illumination, low contrast, noise, and irregularity of the objects. Segmentation refers to partitioning an image into different parts or regions based on similar characteristics in neighboring proximity.

Deep learning systems, as a prominent segment of the rising artificial intelligence (AI) technology in recent years, have been reported with significantly improved diagnostic accuracy in medical imaging [11, 17]. These intelligent systems are aiding an accelerated progress in early-stage diagnosis and treatment of many diseases including automatic detection of the liver, lung, and brain diseases [16]. Therefore, the aim of our study is to develop a deep learning model for automatic diagnosis of regions of the lungs infected with the COVID-19 virus using chest CT volumes.

Minaee et al. [18] investigated the application of deep learning structures on chest radiography images to detect COVID-19 patients. For this purpose, they employed four popular convolutional neural networks, including DenseNet-121, ResNet18, SqueezeNet, and ResNet50 to identify COVID-19 disease in the analyzed chest X-ray images. Also, transfer learning on a subset of 2000 radiograms was applied to all networks to overcome the overfitting problem and improve the models' accuracy. Fan et al. [14] applied a lung infection segmentation deep network (Inf-Net) for segmenting the infected tissue in a CT slice automatically. In the first step, a parallel partial decoder is employed for aggregating the high-level features and creates a global map. Then, to increase the accuracy, the implicit reverse attention and explicit edge-attention were incorporated into a model to segmentation the boundaries.

A 3D deep convolutional neural network (DeCoVNet) proposed in [4] for detecting COVID-19 from CT volumes. They used a pretrained UNet model to generate the 3D lung masks. The proposed DeCoVNet was divided into three stages. The first stage is called the network stem, which consisted of a vanilla 3D convolution. A batch normalization layer and a pooling layer with a kernel size of 5 × 7 × 7 were used to preserve rich local visual information based on the ResNet [19] and AlexNet [20]. Also, two 3D residual blocks (ResBlocks) were employed in the second stage. Lastly, a progressive classifier (ProClf) was utilized.

Early-phase detection of Coronavirus proposed by [21] which employed five different feature extraction algorithms. To classify the extracted features, support vector machines (SVM) along with 10-fold cross-validation during the classification process were applied.

To overcome the limitations of previous works, a new hybrid algorithm for finding the location and boundary of the infected tissue from clinical CT images which takes advantage of clustering, local descriptor, and convolutional neural network is introduced. It is broadly considered to be challenging to find the exact location of the lesions inside the lung and extract their borders precisely due to the impact of the COVID-19 which caused the much similar intensity values across the lung. The growing progress of deep learning in all areas of image processing was a great motivation for this study. This work is interested to investigate the power of a CNN model for detecting and segmenting the infected regions inside the lung due to the COVID-19.

## 2. Methodology

**Figure 1:**
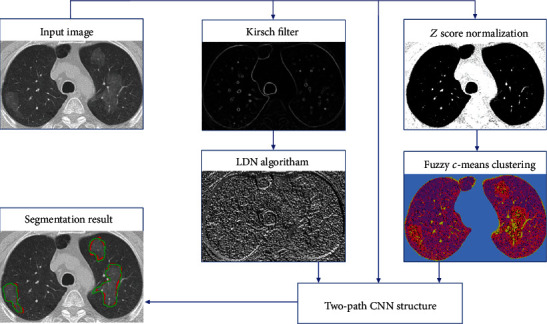
Schematic of the proposed pipeline for segmentation of the infected tissues.

The remaining parts of this paper are organized as follows. In Section 2.1, the *Z* score normalization technique is represented. In Section 2.2, the fuzzy clustering method is described. In Section 2.3, a local directional number patterns (LDN) encoding approach is proposed. In Section 2.4, the architecture of the convolutional neural network (CNN) is demonstrated. In Section 2.5, our CNN pipeline is represented. The explanation of the dataset, evaluation metrics, and experimental results are clarified in Section 3. Our algorithm is displayed in Figure 1.

### 2.1. Image Normalization

As indicated in [22], due to the presence of the statistical noise in the computed tomography images (CT images), a deviation in the Hounsfield units (HUs) about a mean can be observed that lead to a high variance in the gray scale or RGB values of all image pixels. These unwanted noises that affect the ability to visualize anatomic structures can be categorized into three main sources: (1) electronic noise that is an unwanted disturbance in an electrical signal caused by electrical equipment in the neighborhood, (2) noise of the reconstruction procedure caused by imperfections in the receiver coils, and (3) stochastic noise.

As the stochastic noise is the principal source of noise in these kinds of imaging, the bad effects can be diminished during the imaging procedure by increasing the amount of photons (by considering a tradeoff between radiation risk and image quality). However, in obtained images from any hospital or medical center, a significant amount of noise is observed which needs to be removed before starting the process of the segmentation.

By further investigation, we found out that a normalization approach can be beneficial to create a smooth image along with increasing the contrast of illumination near the border of the organs. So, to overcome the mentioned problems and enhance the result of the segmentation, a *Z* score normalization technique is employed so that all the nonzero values inside the image have a unit variance and zero mean ([23–25]; Jafarzadeh [26]). Equation (1) outlines how to apply *Z* score normalization.

(1) $$\begin{array}{c} Z=\frac{\left(x-\mu \right)}{\sigma } \end{array}$$

where *σ* and *μ* indicate the standard deviation and mean value of nonzero pixels, respectively. Moreover, *x* describes the intensity of the current pixel.

**Figure 2:**
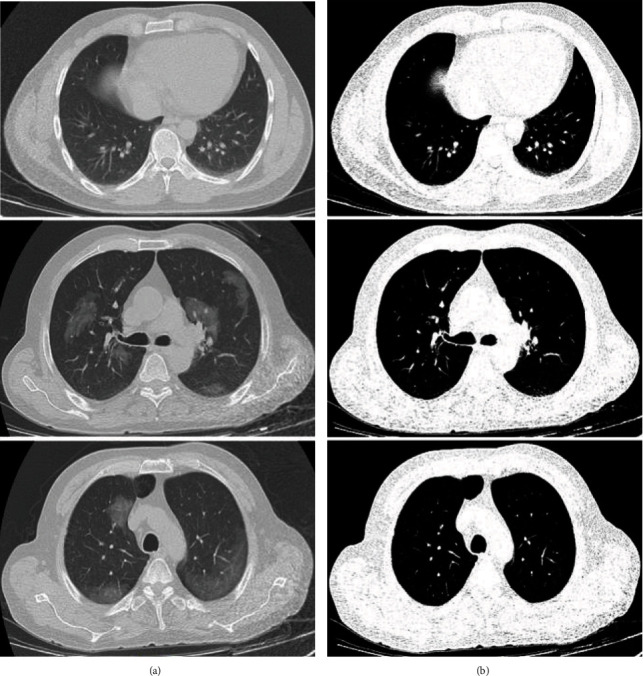
A demonstration of employing *Z* score normalization approach. (a) Original images. (b) *Z* score normalization.

The outcomes of the normalization strategy are depicted in Figure 2. In Figure 2, the first column shows the chest CT images of patients, and their corresponding lesions in the second column demonstrates the *Z* score output. As illustrated in Figure 2(b), the borders of both the lungs are detected exactly without the effect of the lesions.

### 2.2. Fuzzy *c*-Means

After detecting the borders of the lungs with high accuracy, we need to recognize the volume and border of the infected areas inside the lungs more efficiently. The image of the detected lungs achieved from the previous stage has to be clustered to segment the infected areas from the other organs (background tissue). Clustering can be outlined as an unsupervised strategy that is aimed at fragmenting the input data (image or signal etc.) into the predefined segments (such as *K*-means method) or automated recognize parts (such as mean-shift method) based on certain criteria such as differences in the color, magnitude, and location [27–30]. The fuzzy *c*-means (FCM) algorithm used in our work is an unsupervised data dividing/splitting strategy. In this method, data is split into *n* predefined natural groupings, namely, the so-called clusters such that every single pixel in the dataset be owned by at least two clusters with dissimilar weights. In this fuzzy partitioning technique, finding the cluster center of each segment and related pixels are accomplished through an iterative optimization of the objective function [31–33]. This iterative optimization is accomplished by minimizing the following membership cost/objective function:

(2) $$\begin{array}{c} E=\overset{m}{\underset{k=1}{\sum }}\overset{N}{\underset{i=1}{\sum }}\mu_{ki}{\left\|{\text{pixel}}_{i}-{\text{center}}_{k}\right\|}^{2}, \end{array}$$

(3) $$\begin{array}{c} \mu_{ki}=1/{\left(\overset{m}{\underset{j=1}{\sum }}=1\left(\frac{{\text{pixel}}_{i}‐\text{cente}{\text{r}}_{k}}{{\text{pixel}}_{i}‐\text{cente}{\text{r}}_{j}}\right)\right)}^{t},\overset{m}{\underset{k=1}{\sum }}\mu_{ki}=1,\mu_{ki}\in \left[0,1\right], \end{array}$$

where center_*k*_ shows the center of the *k*th cluster and pixel_*i*_ illustrates the *i*th sample of *I*, *μ*_*ki*_ outlines the membership value of the *i*th sample with respect to the *k*th cluster which is linked inversely to the distance from pixel_*i*_ to the cluster center center_*k*_, *m* defines the number of clusters, *t* refers to the level of cluster fuzziness, and *N* denotes the number of pixels image pixels *I*.

**Figure 3:**
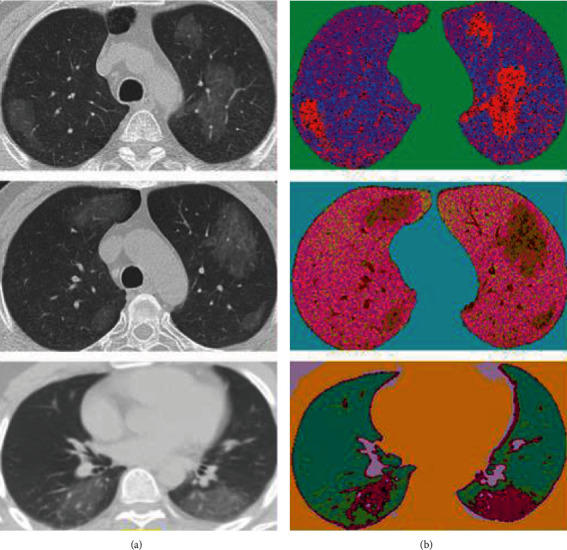
A demonstration of employing fuzzy *c*-means clustering technique. (a) Original images. (b) Clustered images. For better understanding, the colors of the clusters are in the RGB domain with random values.

**Figure 4:**
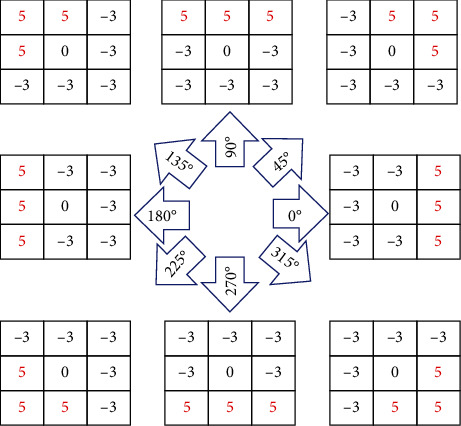
Nonlinear Kirsch kernels in 8 rotations [15].

The result of the clustering on the lung's image is represented in Figure 3. For better visualization, we applied a random value to each cluster in the RGB domain. As is illustrated clearly, by defining the number of five for the center of clusters by experimental results, a high distinction between the lesion and normal tissue can be observed in many samples. It means the number of clusters more or less than five cannot obtain an acceptable result. However, as depicted in Figure 4, in some CT images due to much color similarity between the normal and lesion tissues, using only a clustering method to segment the lesions is not optimal. So, in the next step, textural analysis approaches will be employed to improve segmentation accuracy as much as possible.

### 2.3. Local Directional Number Pattern

Textural analysis of medical and biological images attempts to mine some characterizations of a surface texture such as smoothness, roughness, contrast, colors, and shapes [34]. As presented in many works [35, 36], numerous types of local descriptors are used for converting images into a new representation based on the predefined coding rules or codebook of visual patterns.

Local ternary patterns (LTP) and local binary pattern (LBP) feature descriptors are easy to implement and be influenced by the change of the pixel intensity of nearest-neighbor (circular, rectangular, etc. neighborhood) in clockwise or counterclockwise to alter (encoding) the low-level information of a spot, edges, curve, and line inside an image and calculate the outcome as a binary value [37, 38]. Owing to the robustness of the gradient value than a gray level intensity in encoding applications, in recent investigations, some techniques based on the gradient value such as local word directional pattern (LWDP) and local directional number patterns (LDN) have attained much attention [36]. The LDN operates in the gradient domain to create an illumination-invariant representation of the image. It uses directional information for recognizing edge locations that their magnitudes are insensitive to lighting variations.

**Figure 5:**
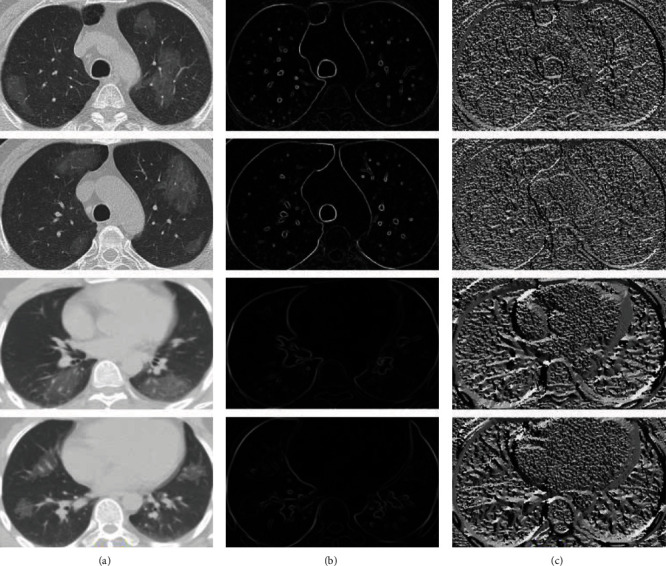
The result of applying the Kirsch filter and LDN approach to a chest image. The second column illustrates edge detection using the Kirsch filter. The third column demonstrates the results of the LDN technique.

In our work, the first phase for encoding the chest images is to define the location and value of all significant edges. This is implemented by operating 8 directions of Kirsch kernels (filters) that are rotated by 45° in 8 main compass directions (Figure 4). These nonlinear edge detector kernels are responsible for identifying the final edges. Each filter produces a feature map, and only the maximum value in each location is selected to create a final edge map [39, 40]. An example of employing the nonlinear Kirsch filter to the chest images is depicted in Figure 5. This section causes a substantial increase in final lesion segmentation, especially when the border of the lesions is vague.

### 2.4. Convolutional Neural Network Design

Automated recognition of patterns in data by computers based on knowledge already obtained is called pattern recognition. It has applications in image analysis, information retrieval, signal processing, bioinformatics, data compression, statistical data analysis, computer graphics, and machine learning [27, 31, 33, 41–44].

In machine learning approaches and applications, the convolutional neural network (CNN) structures demonstrate a high capability to extract and classify some key features and bridging the gap between the capabilities of machines and humans [45–47]. The structure of a CNN was inspired by the organization of the visual cortex in the human brain and is similar to that of the connectivity pattern of neurons. Every neuron responds to an irritant only in a constrained region of the visual field known as the receptive field. The CNN structure that is originally designed for image analysis largely exploits the low level and high level of the textural features and is used in many applications including action detection and automated lesion segmentation [48, 49].

This neuron-based pipeline that captures temporal and spatial dependencies has a grid-like topology and permits us for extracting characteristics powerfully from the 1D or 2D input data by passing through a stack of convolution layers with the predefined dimension of the filters [36, 50, 51]. This grid-like model is a class of deep learning networks and has numerous trainable biases and weights based on the type of the topology and is applied for feature extraction, regression, and classification. These trainable weights need to be defined randomly at the beginning.

This structure is able to extract high-level features automatically from raw input features, which are considerably powerful than human-designed features. The core building block of a CNN is outlined as the convolutional layer which calculates the dot product between input data and a set of learnable filters, much like a traditional neural network [49, 52–54]. It should be noticed that the dimension of the filters is smaller than the dimension of the input data [49, 55]. The computed feature maps using the convolutional layer are achieved by stacking the activation maps of all kernels along the depth dimension. The output of one kernel (filter) applied to the previous layer is called the feature map. In the convolving process, for controlling the dimension of the feature maps, padding the input data with zeros around the border can be employed.

Mostly, the spatial-temporal dependencies at various scales are able to be effectively obtained by the convolutional layers. The dimension of the kernel which defines the dimension of the receptive field needs to be selected based on the depth of the applied 1D, 2D, or 3D data. Also, stride defines how much the convolution filter can be moved at each step. Moreover, the bigger strides lead to less overlap between the receptive fields (smaller feature map) [55].

The high-level features are extracted (such as the hand, legs, and, body in pedestrian detection) in the deeper convolutional layers of the model, while the first convolutional layers are responsible for mining the low-level information including curves, edges, and points. It should be mentioned that the numbers of columns and rows for each filter need to be an odd number, for instance, 9 × 9, 7 × 7, and 3 × 3 [54].

It is noteworthy that the dimension of the extracted features in the last convolutional layer is greatly smaller than the input matrix (1D or 2D matrix). The diminution in the width and height of the image relies upon the length of the strides and the filter size employed for the convolution procedure.

The output of the convolution layer is fed to the activation layer in order to help the network learn complex patterns [56]. This layer leaves the size of the applied matrix (data) unchanged. To decrease the consequence of the vanishing gradient in the training process, an activation function is utilized for each feature map to improve the computational effectiveness by inducing sparsity [55, 57].

In this study, the nonlinearity (ReLU) activation function has been employed to shift the negative values to zero. The ReLU act as a linear function for the positive and zero values. As all negative values change to the zero number, it leads some nodes to completely die and not learn anything. It means fewer neurons in the model would activate because of the limitations imposed by this layer.

Some of the most important benefits of the ReLU layer can be expressed as follows [58–60]:

1. Train deep networks: the architecture with large labeled datasets is able to reach the best performance on purely supervised tasks
2. Linear behavior: the procedure of decreasing the cost function (optimization) in the CNN is much easier if their behavior could be close to a linear manner
3. Representational sparsity: as the ReLU layer shift the negative input values to the zero values, it causes some of the neurons in the hidden layers in neural networks to have zero values. In other words, by removing the effect of some neurons with zero weight, an accelerating in the learning process can be achieved which is called a sparse representation
4. Computational simplicity: dissimilar to the tan*h* and Sigmoid activation functions, ReLU consists of only simple operations in terms of computation so that computing the exponential function in activations can be eliminated and therefore much more practicable to implement in models

The ReLU layer does not cause the vanishing gradient problem and avoid easy saturation. Also, due to overcoming the vanishing gradient issue, models are permitted to learn faster and perform better. Equation (2) outlines how the ReLU activation function accomplishes [58, 59].

(4) $$\begin{array}{c} f\left(x\right)=\max\left(0,x\right), \end{array}$$

where *x* demonstrates the input value and *f*(*x*) is its related output.

Since in object recognition applications, there is evidence that demonstrates the form, dimension, color, or position of the object has no matter, only the spatial variances need to be investigated. In order to accomplish this, a downsampling layer is applied by summarizing the key information in patches of each feature map without losing any details that lead to a good classification. In contrast to the convolution operation, the pooling layer has no parameters and only slides a window over its input, and simply takes the predefined value (mean, max, etc.) in the window. Furthermore, as the quantity of pixels in this layer (in both row and column) is dropped, it leads to shortening the training time and combats overfitting [54, 61–63].

An appropriate technique for dimensionality reduction of feature maps is to reduce the number of parameters and computation in the network so that the model can be robust to alter the high-frequency information (key information) and preserves vital features [55]. This dimension-reduction procedure happens by utilizing a filter along the spatial dimensions (width, height) with a predefined dimension. This layer is regularly incorporated between two sequential convolutional layers. The max pooling layer accomplished in this study first partitions the extracted matrix of features into a set of parts with no overlapping and then takes the maximum number inside each district. The max pooling strategy also employs as a noise suppression technique [53, 64].

In a CNN structure (shallow or deep CNN), since the receptive field in the last convolutional layer does not cover the entire spatial dimension of the image, the generated features by the last convolutional layer correspond to a section of the input image. Therefore, one or some FC layers are obligatory in such a scenario. A fully connected layer (FC) allows the model to learn the nonlinear combinations of the high-level features in an input image.

Each node in the fully connected layer produces a single output with its learnable corresponding weight that is linked to all the activations in the previous nodes [56]. It is noteworthy that before applying the generated feature matrixes to the fully connected layer, all 2D features have to be changed into a one-dimensional matrix (1D vector) [65–67]. The latest layer for classification tasks in a CNN-based pipeline is the Softmax regression layer which is able to differentiate one from the other. The Softmax regression is also called multinomial logistic, multiclass logistic regression, or just maximum entropy classifier. This single-layer regression tries to normalize an input value into a vector of values to demonstrate how likely the input data belongs to a user-defined class. Also, as the output values are between the range (0, 1), the sum of the output values obtained from the probability distribution procedure is equal to one [52, 53, 67, 68].

For the training step, since we are not working with a big dataset with hundreds of different samples from many patients, it is enormously easy for the CNN-based models to converge or to be specialized according to its reliability level and application area (to be less intelligent). To overcome this issue, there are two main strategies: (1) transform learning and (2) data augmentation.

The transform learning method is utilized to bring some trained biases and weights into any pipeline rather than select them randomly at the first step. Data augmentation is a popular method for artificially boosting the number of training examples [69, 70].

### 2.5. Our CNN Pipeline

**Figure 6:**
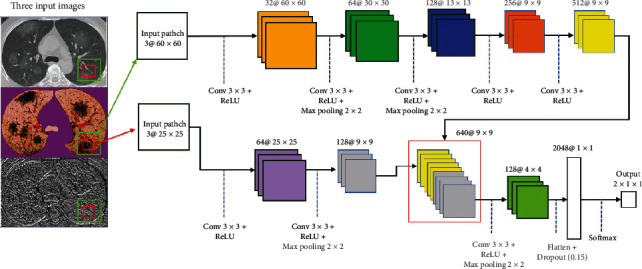
Our implemented two-path CNN model using three distinct inputs.

As mentioned before, CNNs are used to explore significant details from an input of raw pixels more efficiently. Hence, in this study, we investigated the probability of the presence of the lesions caused by CVOID-19 using a novel model based on the combination of global and local features. Moreover, to maximize the segmentation accuracy for even small damaged healthy tissue, the proposed approach concludes three distinct input images instead of a single one. The three input images include original image, fuzzy clustered image, and encoded image (LDN). These three different inputs enable our model to handle many types of variability in the raw input pixels. The flowchart of the proposed structure is shown in detail in Figure 6.

When we use CNNs for automatic feature extracting that are effective for various tumor or lesion detection problems, the need for preprocessing and highlighting the suspect regions is significantly reduced. This is due to the fact that the CNN-based structures have millions of parameters that are able to produce the best suited feature maps for expressing the class probability. Although numerous CNN pipelines have been recommended for lesion segmentation in recently published papers, none of them has concentrated on applying the combined the textural encoding algorithm, fuzzy clustered, and raw image pixels as an input to a CNN structure. Since miscellaneous texture or images definitely encompass complementary and detailed information (features), our experimental outcomes for small samples (data) imply that this complex two-path strategy is effective to enhance the score of the evaluation indexes.

While analyzing the complex texture of our input images, due to many similarities of the lesion (infected area) with normal tissue in the margin of the lesion, semiglobal and local features must be taken into account. Moreover, the lesions may appear anywhere on the lung since COVID-19 has a multifocal distribution that for gaining better results need to have knowledge of neighbor information in a little further of each analyzing pixel location. As is clearly indicated in Figure 6, the recommended cascading model is based on investigating key features using two distinct local and global paths.

In contrast to some other recently published methods such as studies by Hu et al. [71], Wang et al. [4], and Fan et al. [14] that employ all pixels inside the image as an input, our method only considers two patches from each applied 2D data (totally 6 patches) as an input to classify each pixel inside the output image. In other words, if there are 1000 pixels inside the image, the number of the produced patches are 1000 × 2, and due to the use of the three input images, there are 1000 × 2 × 3 patches. This is very interesting that using both local and global patches with a different route for extracting features can get better results compared to using only one of them.

In our model, two distinct routes are employed; the first one (upper path) comprises of the five convolutional layers for extracting the global features. The other path (bottom path) utilizes two convolutional layers for extracting the local features. The local and global investigation windows (patches) are 25 × 25 and 60 × 60, respectively.

The semiglobal patches are employed for providing key details about the analogous touching textures with scar tissues, while the local patches are applied more for recognizing inflammation in the tiny air sacs. Moreover, the outcome of our strategy for inflammation detection highly depends on information extracted from the global windows. In Table 1, we exhibit the effect of employing semiglobal and local patches in the ultimate outcome of our approach. As is depicted in Table 1, the best observed Dice score is obtained when the sizes of the local and global patch are 25 × 25 and 60 × 60, respectively.

The size of the local region is 25 × 25 × 3, which three implies three distinct input images. The selected regions are convolved using 64 kernels to generate the feature maps based on the 3 × 3 receptive field. In the next layer, the number of filters is changed to 128 with the same receptive field. After producing feature maps in the first layer, the max pooling layer is not, while after the second layer, max pooling decreases the dimension of the produced feature maps.

Unlike the local features extraction path, in the global feature extraction procedure, five convolutional layers are employed. In this path, only two intermediate layers are employed that are using the max pooling approach. All extracted feature maps with the size of 9 × 9 at the end of each route are concatenated to create 384 feature maps in order to use in the next convolutional layer. After the concatenation step, 128 kernels are applied to these feature maps, and then, a max-pooling layer changes the all dimensions to the 4 × 4. Then, all created feature maps are transformed into a 2048 × 1 feature vector. Lastly, by applying a Softmax layer, all extracted data are tagged to one of two expected classes (1 implies the inflammation and 2 shows the normal tissues.).

For minimizing the cross-entropy loss, the proposed CNN structure with two routes was learned through stochastic gradient descent (SGD) in 1000 epochs with a batch size of 128 [72], in Equation (5). Our pipeline calculates the discrepancy between the predicted output and groundtruth for lesion segmentation. The dropout is applied before the FC layer, which is aimed at avoiding “overfitting” and equals to 0.2. For optimization, we applied a weight decay of 0.0001 and a learning rate of 0.01. In the output layer, two logistic units to obtain the probabilities of the given sample belonging to either of the two classes were employed. The backpropagation scheme was applied to generate the derivative of the objective function.

(5) $$\begin{array}{c} {\text{loss}}_{i}=-\log\left(\frac{e^{U}k}{\sum_{d=1}^{L}e^{U}d}\right), \end{array}$$

where loss_*i*_ implies the loss value for training data *i*, and *U*_*K*_ demonstrates the raw production score (is not normalized) for the reference class *K*.

The unnormalized production score is generated by multiplying the outputs from the previous FC layer with the parameters of the corresponding logistic unit. To find the normalized scores for each class between 0 and 1, the denominator aggregates the scores for all the logistic units *L*. Since two output neurons are presented at the output layer, in the above equation, *L* is equal to 2.

## 3. Experiments

### 3.1. Datasets

The proposed novel technique and three recently published models were investigated on a public chest dataset [73] to evaluate the reliability, validity, and accuracy of experiments. This dataset is available at https://github.com/UCSD-AI4H/COVID-C. To segment the corrupted tissues accurately, four experienced specialists segmented the borders manually. It is noteworthy that by employing an augmentation strategy to increase the number of data, a lot of new samples are generated. Also, 70% of data for training, 10% for validating, and 20% for testing are used. Data augmentations are useful approaches to decrease the validation and training errors. The augmentation methods artificially inflate the training dataset size by either data oversampling or warping. When in the augmentation process, the labels of the existing images are preserved; this process is called data warping augmentations. This method includes augmentations such as color and geometric transformations, adversarial training, random erasing, and neural style transfer. Oversampling augmentations generate synthetic samples and add them to the training set [74].

Six approaches of data augmentation are utilized in this paper to increase efficiency, namely, flipping, color space, rotation, translation, noise injection, color space transformations, and random erasing.

In flipping, a horizontal axis flipping is used. In the color space, contrast enhancing is employed. In rotation, 180 degrees is selected. In translation, left, right, up, and down are applied. In noise injection, a Gaussian distribution is utilized. In the color space transformations, decreasing and increasing the pixel values by a constant value are applied. In random erasing, an *n* × *m* patch of an image is randomly selected and masking it with zero values.

### 3.2. Evaluation Metrics

In this study, the following nine measures were calculated by comparing the segmentation results with that of lesions segmented by the experts to appraise the proposed architecture's efficiency. The promising accuracy of the proposed two-path architecture was assessed using recall, precision, *F* score, ASD (average surface distance), RVD (relative volume difference), RMSD (root mean square symmetric surface distance), MSD (maximum surface distance), VOE (volume overlap error), and DICE (Dice similarity) [15, 75–77]. Some mentioned metrics are defined as follows:

(6) $$\begin{array}{c} \left\{\begin{array}{c} \text{Precision}=\frac{\text{TP}}{\text{TP}+\text{FP}}\times 100\%,\\ \text{Recall}=\frac{\text{TP}}{\text{TP}+\text{FN}}\times 100\%,\\ \text{F}=\frac{2\times \text{Precision}\times \text{Recall}}{\text{Precision}+\text{Recall}}\times 100\%,\\ \text{DICE}=\left(2\times \frac{\text{TP}}{2\text{TP}+\text{FP}+\text{FN}}\right)\times 100\%,\\ \text{VOE}\left(M_{s1},M_{s2}\right)=\left(1-\frac{M_{s1}\cap M_{s2}}{M_{s1}\cup M_{s2}}\right)\times 100\%,\\ \text{RVD}\left(M_{s1},M_{s2}\right)=\left(\frac{M_{s1}-M_{s2}}{M_{s2}}\right)\times 100\%,\\ \text{ASD}=\;\frac{1}{\left|B_{M_{s1}}\right|+\left|B_{M_{s2}}\right|}\times \left(\underset{x\epsilon B_{M_{s1}}}{\sum }d\left(x,B_{M_{s2}}\right)+\underset{y\epsilon B_{M_{s2}}}{\sum }d\left(y,B_{M_{s1}}\right)\right), \end{array}\right. \end{array}$$

where *M*_*s*1_ and *M*_*s*2_ denote the result of segmentation using our strategy and ground-truth mask, respectively. Also, *B*_*M*_*s*1__ and *B*_*M*_*s*2__  imply the borders result of our segmentation technique and ground-truth image, respectively. Moreover, the FN, FP, and TP represent false negative, false positive, and true positive, respectively [37, 78].

Dice similarity coefficient (DSC) is defined as one for a perfect segmentation and is a statistical tool for measuring the similarity between two sets of data. MSD measures the distance between the borders of each segmented object from its corresponding border in the groundruth image. Measuring the difference between the segmented object and related object in the groundtruth image can be calculated by RVD, in which the positive value implies oversegmentation and the negative value represents the undersegmentation result. It means that the best value is zero that indicates the segmented object is equal to the groundtruth image.

### 3.3. Experimental Results

Our two-path architecture was implemented in Python, and the experiments were run on an Intel(R) Core(TM)i7-3.4 GHz + GEFORCE GTX 1080 Ti GPU+16 gigabytes of RAM under the windows 10 (64-bit) operating system. The results of our pipeline using 3 distinct input images were appraised utilizing the corresponding ground-truths and reported in Tables 2 and 3. In our dataset samples with a large diversity in the volume of the lesions, not well-defined borders (unclear or blurred margin) have the greatest part of the train, validation, and test samples.

**Figure 7:**
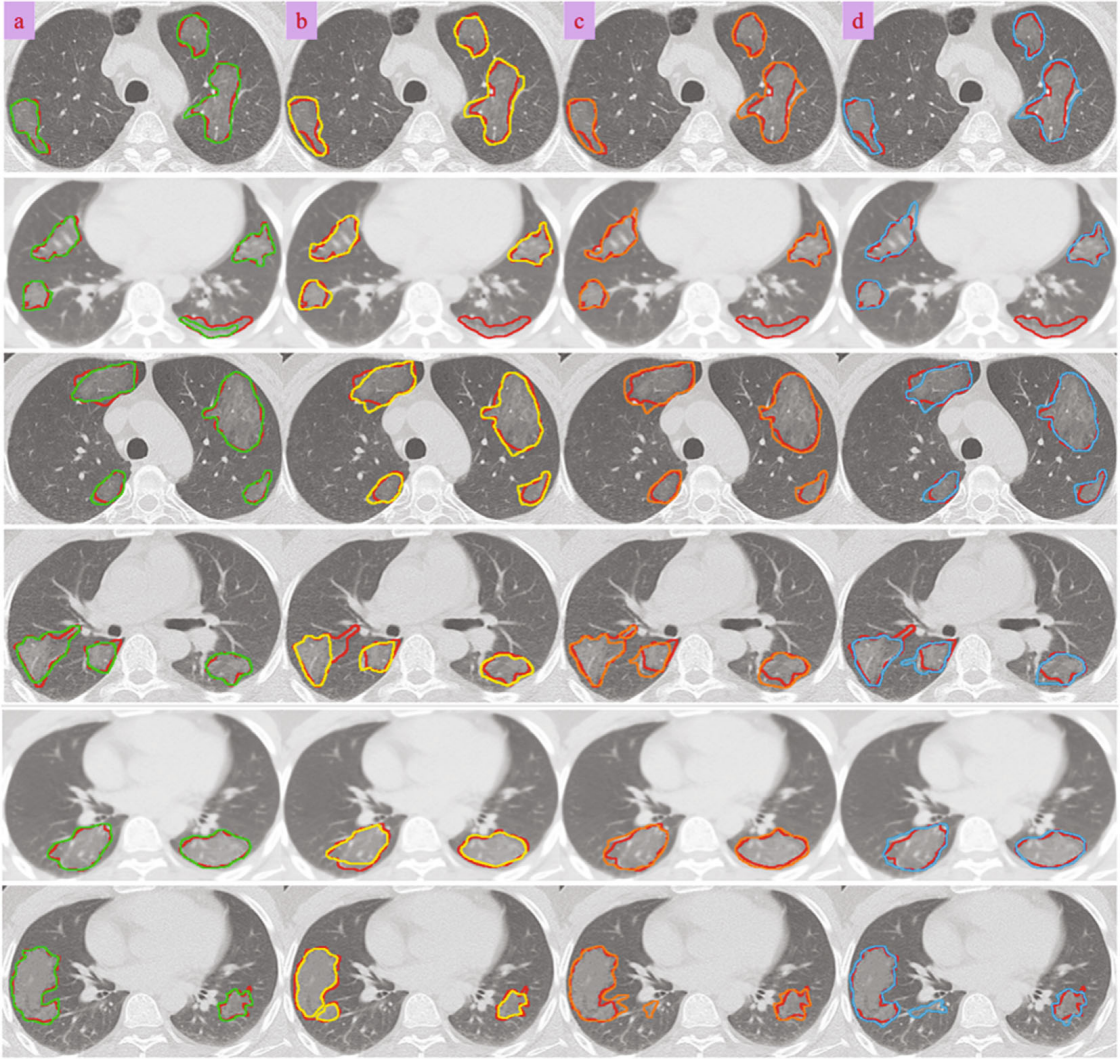
Comparisons between four different kinds of strategies for COVID-19 infection detection. The red contours indicate the reference border (groundtruth). Segmentation based on the (a) proposed strategy, (b) DenseNet201 [1], (c) weakly supervised deep learning [71], and (e) weakly supervised framework [4].

For exemplifying the significance of utilizing the grouping of the LDN encoding approach, *Z* score normalization technique, and CNN framework to accurate estimating borders, Figure 7 demonstrates the outcomes of our structure (drawn by a green line). The results of our method compared to three other recently published methods are shown in Figure 7 on a few slices with the intensity inhomogeneity, ambiguous boundaries, heterogeneous appearances, and various infection shapes. Accordingly, it can noticeably be observed that the intensity inhomogeneity and ambiguous boundaries inside the lung due to the infection cause the infected regions are not suitably extracted when the DenseNet201 [1], weakly supervised deep learning [71], and weakly supervised framework [4] approaches are applied.

As indicated in Figure 7, segmentation by employing the DenseNet201 [1] structure shows the fewest match with the reference data (groundtruth), especially when similar intensity values are encountered near the borders of the infected regions. Weakly supervised deep learning [71] is good to recognize the infection boundary when there is much distance (more than 20 pixels) between two lesions, but when in the small distance (less than 20 pixels), it performs so poorly and the chance of combining two lesions is highly increased. Also, the DenseNet201 [1] method undersegment the infected areas in the most cases, whereas the weakly supervised deep learning [71] and weakly supervised framework [4] models oversegment with equivalent intensity values. Moreover, such pipelines are more prone to boundary leakage, especially when there are unclear borders among the different kinds of infection progress. To solve this issue, we came up with the idea of employing both local and global features when there are three representations of the infected and noninfected tissues. Our model also has not noteworthy boundary leakage, substantial oversegmentation, or undersegmentation, predominantly in particular sections that are near the white objects. By using the *Z* score normalization and fuzzy clustering methods, our approach is more capable of enhancing the contrast near the border of the lung to obtain more accuracy in the distinction of an infected region and vague border of the lung. Considering the heterogeneous textures, opaque appearance of the infected tissue, misalignment of the infection boundaries, unclear borders, and different dimensions of the infection regions, it is more evident that our pipeline suitably finds a pattern most similar to the infected area, which demonstrates its robust performance under realistic scenarios on countless infection outlines. It worth mentioning that in all methods, the white tissue (pulmonary nodules) near the infected area cannot be properly recognized due to much similarity between both tissue values. The results may get better if the amount of training data is increased.

**Figure 8:**
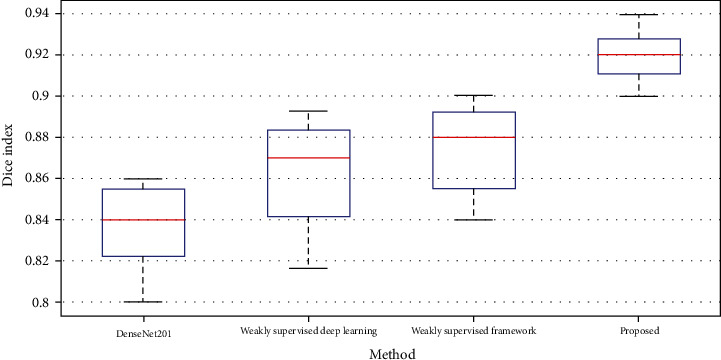
Comparison between the Dice scores of the four models employed for lung infection segmentation in CT images.

The proposed two-path CNN structure achieved a higher segmentation performance than the other three evaluated methods when other representations of the lung images are applied; meaning more substantial features are available to achieve the best distinction between classes. The efficiency of our technique on different CT infected lungs was assessed using the Dice similarity index, as illustrated in Figure 8. The Dice score averages for the segmented infection areas with diverse appearance varied from 80% to 94%. As is shown, the worst result belongs to the DenseNet201 approach with an average of 84%. The result of our approach implies that the appearance, intensity values, and outline of the infected tissue cannot significantly affect the segmentation performance and efficiency.

Tables 2 and 3 indicate the comprehensive evaluation of our complex strategy for lesions segmenting and compare it with the results of other mentioned methods on our dataset.

Table 2 implies a quantitative comparison, in practice, between the automated lesion segmentation outcomes of the novel proposed two-patch model over the other three mentioned approaches. For each index in Tables 2 and 3, the highest values of RVD, ASD, RMS, MSD, VOE, recall, precision, and *F* score are highlighted in bold. The outcomes of every first five assessment criteria are demonstrated by standard deviation and mean values in Table 2. The proposed two-route segmentation model gains a smaller mean in mentioned assessment criteria. The obtained VOE is meaningfully altered between all appraised architectures, while the outcomes of RMS and ASD imply the lowest variance. The RVD score for DenseNet201, proposed CNN, and proposed CNN+fuzzy *c*-means algorithms are less than 0. Also, adding the LDN method to the proposed CNN model leads to observe the positive value in the RVD result. The RMS score imply that the proposed CNN+fuzzy *c*-means+LDN and proposed CNN+LDN methods produced the best outcomes among the seven structures. Also, the DenseNet201 technique gains the highest mean score of RMS.

In addition, the mean value of MSD and VOE of the models employed by DenseNet201 and weakly supervised framework were outstandingly higher as compared to our outcomes. Moreover, both the weakly supervised deep learning and the weakly supervised framework models show a large standard deviation in the RVD; however, a major standard deviation in MSD score is obtained in the DenseNet201 method. The observed results in the ASD and VOE indicate that adding LDN and fuzzy clustering methods to our CNN model can significantly improve our model accuracy.

The results in Table 3 indicate the measurements for differentiating the objects inside the lung, including normal and infected tissues. As can be observed in Table 3, our technique, CNN+fuzzy *c*-means+LDN, consistently performs the best among all approaches. The *F* score, precision, and recall of the DenseNet201 and weakly supervised deep learning structures are highly similar to the proposed CNN algorithm; however, by adding the LDN or fuzzy clustering approach, these three criteria are highly increased. Also, the DenseNet201 approach gains the worst results and our architecture obtains the competitive performance on lesions segmentation in all evaluation metrics.

## 4. Discussion and Conclusions

In this study, we implemented a two-path CNN pipeline that incorporates the three distinct input images, to automatically segment the infected tissues inside the lung caused due to the COVID-19 from CT images. For a better demonstration of the tissues to extract more key features inside the CNN model, we showed the input CT image represented in the two other different ways which each of them includes some unique information. Due to inflammation inside the lung because of COVID-19, infected areas near the border of the lung are highly difficult to segment. So, our algorithm first employed a *Z* score normalization technique to obtain a more distinguishable lung border from the original image. Then, by using a fuzzy clustering method, all tissues in the image are clustered and obtain a distinct pixel value for all pixels corresponding to each cluster. This approach helps the CNN pipeline for decreasing the convolutional layers for extracting some key features and leads to a drop in the training time of the pipeline and increase the final efficiency.

Then, an LDN encoding approach was implemented for representing the information of the images in another form to extract more essential details from the input image. This strategy roots in the fact that sometimes by changing the representation domain (like frequency domain rather than the time domain) some other substantial features can be observed.

We also represented a new two-route CNN model that considered semiglobal and local information to categorize each pixel in the input image to one of the two normal and infected tissues. The number of the convolutional layers in the global route is more than the local route, while the kernel size for all convolutional layers is the same. To overcome the overfitting problems and boost efficiency, using data augmentation methods, the number of samples has been increased. Lastly, using the CT image and two obtained images, our CNN structure was trained.

The suggested two-route segmentation pipeline was appraised on a public dataset which 70% of data for training, 10% for validating, and 20% for testing were used. Our significant findings demonstrate that our CNN pipeline and three distinct input images gained the following: (1) acceptable performance even if the infected area shared an extended border with touching tissues, (2) appropriately robust as indicated by the negligible standard deviations which show the uniformity of the values for all the nine criteria, and (3) accomplished well in the detection and segmentation process even for the intricate cases with numerous unalike categories of the infection, which had the amoeboid shapes and analogous thicknesses.

The proposed architecture satisfactorily overcomes the difficulty of failing in accurate detection of the lesions at the presence of the similar adjacent tissues and identification of an uneven border where it seemed to not properly appear to exist with an aim to reach superior outcomes. In addition, the employed technique does not require more extra parameters for feeding into the algorithm apart from one CT image to define the position of the lesions and border detection. But the functional limitation of this architecture is that the white matter (pulmonary nodules) inside the normal lung near the border of a lesion cannot properly be recognized from the infected tissue. We think that by increasing the training samples this problem can be solved.

Tables 2 and 3 approve that our technique divides erratic and wide infections and irregular shapes. Most of the segmentation strategies that merely rely on measuring the illumination, energy, thickness, location, and shape could fail when the infected tissue and other touching objects have an analogous density and intensity levels. Under such specific circumstances, applying additional distinguishable features from different kinds of images may result in improving the ability of segmentation and fulfilled a leading role in gently separating infections associated with the abovementioned problems. Our unique pipeline could potentially be more advantageous when encountering diverse infections with the blurred boundaries and wide-ranging lesion sizes. The implemented procedure proposed herein yields a more classification efficiency in terms of simplicity, stability, and time consumption compared to the baseline models.

## Figures and Tables

**Table 1:** Investigating the accuracy of employing dissimilar dimensions of the regions in the final result of the approach.

Size of the semiglobal patch	Size of the local patch	DICE value for lesion segmentation
40 × 40	11 × 11	24%
50 × 50	11 × 11	31%
60 × 60	11 × 11	33%
70 × 70	11 × 11	40%
80 × 80	11 × 11	41%
40 × 40	15 × 15	61%
50 × 50	15 × 15	70%
60 × 60	15 × 15	72%
70 × 70	15 × 15	73%
80 × 80	15 × 15	81%
40 × 40	21 × 21	74%
50 × 50	21 × 21	76%
60 × 60	21 × 21	81%
70 × 70	21 × 21	88%
80 × 80	21 × 21	91%
40 × 40	25 × 25	56%
50 × 50	25 × 25	73%
60 × 60	25 × 25	92%
70 × 70	25 × 25	89%
80 × 80	25 × 25	87%

**Table 2:** Quantitative comparison of infected tissue segmentation outcomes based on our model and three recently published structures. The evaluations are based on average surface distance (ASD), relative volume difference (RVD), Volume overlap error (VOE), root mean square symmetric surface distance (RMS), and maximum surface distance (MSD).

Approach	ASD (mm)	VOE (%)	RVD (%)	MSD (mm)	RMS (mm)
DenseNet201 [1]	5.4 ± 0.3	11.4 ± 7.3	−4.2 ± 5.9	23.6 ± 7.1	5.9 ± 0.4
Weakly supervised deep learning [71]	5.1 ± 0.4	11 ± 7.3	7.8 ± 10.3	21 ± 6.6	5.5 ± 0.7
Weakly supervised framework [4]	6.1 ± 0.6	11.7 ± 4.2	8.3 ± 6.6	22.7 ± 5.2	5.8 ± 0.5
Proposed CNN	6.3 ± 0.5	11.9 ± 6.8	−5.8 ± 3.5	21.3 ± 6.1	5.7 ± 0.4
Proposed CNN+LDN	5.1 ± 0.1	8.3 ± 4.7	6.5 ± 4.1	15.4 ± 4.8	4.7 ± 0.2
Proposed CNN+fuzzy *c*-means	5.5.3 ± 0.4	8.9 ± 5.2	−6.9 ± 7.3	16.5 ± 4.9	5.2 ± 0.5
Proposed CNN+fuzzy *c*-means+LDN	2.8 ± 0.3	5.6 ± 1.2	3.7 ± 5.6	7.4 ± 7.3	3.6 ± 0.2

**Table 3:** Quantitative comparison of infected tissue segmentation outcomes based on our pipeline and three recently published structures. The evaluations are based on recall, precision, and *F* score.

Approach	Precision (%)	Recall (%)	*F* score
DenseNet201 [1]	86%	89%	87%
Weakly supervised deep learning [71]	88%	90%	89%
Weakly supervised framework [4]	91%	89%	90%
Proposed CNN	88%	89%	88%
Proposed CNN+LDN	93%	91%	92%
Proposed CNN+fuzzy *c*-means	92%	94%	93%
Proposed CNN+fuzzy *c*-means+LDN	**96%**	**97%**	**97%**

## Data Availability

The data used to support the findings of this study are included within the article (https://github.com/UCSD-AI4H/COVID-CT).
